# Report of Mortality in Buffalo for the Month of Oct., 1856

**Published:** 1856-12

**Authors:** C. L. Dayton

**Affiliations:** Health Physician


### ART. VI.—*Report of Mortality in Buffalo for the month of Oct.*, 1856.

By C. L. Dayton, M. D., Health Physician.

| DISEASES. | No. | Males. | Females. |
|---|---|---|---|
| Accidens, | 4 | 4 | |
| Asphyxia Immersorum, | 7 | 7 | |
| Apoplexia, | 1 | 1 | |
| Cynanche Trachealis, | 6 | 5 | 1 |
| Cholera Infantum, | 3 | 1 | 1 |
| Cyanosis, | 1 | | 1 |
| Carditis with Hypertrophy, | 1 | 1 | |
| Dentitio, | 3 | 2 | 1 |
| Debilitas Senilis, | 6 | 3 | 3 |
| Diarrhœa, | 4 | 3 | 1 |
| " Chronica, | 1 | 1 | |
| Dysentery, | 10 | 3 | 7 |
| Epilepsy, | 1 | | 1 |
| Enteritis, | 1 | | 1 |
| Eclampsia, | 12 | 4 | 8 |
| Fracture of Spinal Vertebræ, | 1 | 1 | |
| Febris Typhus, | 2 | | 2 |
| " Scarlatina, | 7 | 1 | 5 |
| " Scarlatina Maligna, | 2 | 1 | 1 |
| " Typhoid, | 4 | 3 | 1 |
| " Billiosum, | 1 | 1 | |
| Gangrene, | 2 | | 2 |
| Hyperæmia Cerebralis, | 2 | 1 | 1 |
| Hydrocephalus, | 2 | 1 | 1 |
| Hæmorrhagia Uterina, | 1 | | 1 |
| Hydrops, | 4 | 3 | 1 |
| Hypertrophy of the Heart, | 1 | 1 | |
| Hyperæmia Pulmonalis, | 2 | 2 | |
| Intemperance and Delirium Tremens, | 4 | 4 | |
| Icterus, | 1 | | 1 |
| Ignotus, | 18 | 6 | 9 |
| Morbus Cerebri, | 1 | 1 | |
| Malformation of the Heart, | 1 | 1 | |
| Mania et Enteritis Chronica, | 1 | 1 | |
| Morbus Gastrica, | 1 | 1 | |
| Marasmus, | 7 | 6 | 1 |
| Narcosis, | 1 | 1 | |
| Osteo Sarcoma, | 1 | | 1 |
| Pneumonitis, | 2 | 1 | 1 |
| Peritonitis, | 2 | 2 | |
| Rheumatism | 1 | 1 | |
| Suicide by taking laudanum, | 1 | 1 | |
| Scrophula, | 3 | 2 | 1 |
| Still-born, | 2 | 1 | |
| Spina Bifida, | 1 | 1 | |
| Summer Complaint, | 4 | 3 | 1 |
| Tuberculosis Pulmonalis, | 22 | 8 | 14 |
| Tabes Mesenterica, | 1 | | 1 |
| Total, | 167 | | |

SEXES.

| | |
|---|---|
| Males, | 91 |
| Females, | 70 |
| Sex not given, | 6 |
| Total, | 167 |

AGES.

| | | | |
|---|---|---|---|
| Still-born, | 2 | Between 10 years and 20 years, | 4 |
| 1 day, | 4 | " 20 " " 30 " | 21 |
| Between 1 day and 30 days, | 7 | " 30 " " 40 " | 13 |
| " 1 month and 6 months, | 17 | " 40 " " 50 " | 13 |
| " 6 months and 12 " | 18 | " 50 " " 60 " | 10 |
| " 1 year " 3 years, | 34 | " 60 " " 70 " | 7 |
| " 3 " " 5 " | 7 | " 70 " " 80 " | 0 |
| " 5 " " 10 " | 7 | " 80 " " 90 " | 1 |
| | | " 90 " " 100 " | 2 |
| Total number under 10 years, | 96 | Total number over 10 years, | 71 |
| Ages not given, | 0 | Total, | 167 |

NATIVITIES.

| | | | |
|---|---|---|---|
| American, (white, 64), colored, 1, | 65 | Prussian, | 0 |
| German, | 42 | French, | 7 |
| Irish, | 4 | Scotch, | 0 |
| English, | 4 | Italian, | 0 |
| Canadian, | 1 | Country not given, | 44 |
| Total, | | | 167 |

*Summer Complaint and Convulsions.*—The value of statistics depends, of course, upon their accuracy. With a desire to assist in making the records of mortality in Buffalo, in future more perfect, permit me to say a few words in regard to the above ailments. Since the first of January, 1856, there have been 116 deaths in Buffalo reported as occurring from summer complaint. In the annual report for the year 1855, the number of deaths arranged under the head of diarrhœa and summer complaint, is 172. In the report for the year 1854, I am unable to find the "Disease." May not any disease that has its origin and termination during the summer months, be termed a summer complaint? If so this term is not indicative of any particular organic lesion, or fanctional derangement. We cannot take it for granted that those cases reported as summer complaint were in reality either case of diarrhœa, cholera morbus, cholera infantum, or Asiatic cholera. If, then, the term summer complaint is attached to a specific disease, and that one which has had its origin in Buffalo since 1854, and has destroyed 288

lives, let us, if possible, inquire into its pathology, and attach to it a more expressive and scientific name, if not one more popular.

It seems as if it was absolutely necessary that some hobby should be selected upon which a portion of those dying must annually ride out of life. Convulsions appears to have been saddled for that purpose. There have been 132 deaths in this city reported as occurring from that "symptom," for the ten months ending Nov. 1st. Of the actual diseases that have caused this large number of deaths, we are forever to remain in ignorance. They were reported chiefly by those whom I will call "outsiders." Whether they were epileptic, or apoplectic convulsions, or convulsions produced by a mechanical constriction of the larynx, or the effects of strychnia, the wound of a nerve, or a thousand other causes that might be enumerated, we are not permitted to know. Add to the above the number that have been reported during the same period from unknown causes, 160, and we have a grand total of 292 deaths in ten months, about which we know nothing more than that they had spasms preceding dissolution. The question to be solved is this, Is there a remedy for this morbid state of vital statistics?

---

*Rigidity of Os Uteri.*—Prof. Brickell, of New Orleans, has been successful in the use of lobelia, according to the plan of Dr. Livezey. In a primipara, forty-one years old, he found, after the labor had continued eighteen hours, that the os was open only to about the size of a half dollar, but as rigid as possible, and rather dry and warm. An infusion of lobelia was prepared, one drachm to the pint. Of this about three ounces were administered, by injection, every fifteen or twenty minutes, and in less than one hour the os was found rapidly dilating and softening. Both the vulva and vagina were in an improved condition. The membranes were ruptured, and in half an hour the patient was delivered.—*Memphis Med. Recorder.*

---

*Reduction of Dislocated Femur.*—The Western Lancet states that Prof. Blackman has succeeded in reducing a femur which had been dislocated six months, by manipulation after the plan of Dr. Reid. The operation was performed while the patient was under the influence of chloroform, but the effect of the chloroform was subsiding, and the muscles beginning to act a moment before the bone slipped into its socket. Our readers will find this method of operating described at page 51, volume iv. Memphis Medical Recorder. It is a rare thing to see a dislocation of this kind, of six months' standing, reduced by any means, much less by mere manipulation.—*Ibid.*

